# Impact of Protease Inhibitor‐Containing Blood Collection Tubes on Targeted Proteomic Profiles of Alzheimer's Disease Plasma Biomarkers

**DOI:** 10.1002/alz70856_107138

**Published:** 2026-01-08

**Authors:** Abdul Razak Monto, Xuemei Zeng, Marissa F Farinas, Jeremy M. Gu, Lamia Choity, Kristine A Wilckens, Thomas K Karikari

**Affiliations:** ^1^ University of pittsburgh, Pittsburgh, PA, USA; ^2^ University of Pittsburgh School of Medicine, Pittsburgh, PA, USA; ^3^ University of Pittsburgh, Pittsburgh, PA, USA

## Abstract

**Background:**

Blood‐based biomarkers (BBMs) are promising for diagnosing Alzheimer's disease (AD). However, a significant challenge related to specimen quality is anticipated as BBMs are integrated into real‐world settings, where access to specialized equipment may be limited. We have previously demonstrated that the BD™ P100 (BD‐P100) blood collection tube, a specialized EDTA tube spray‐coated with protease inhibitors, effectively stabilizes classical AD BBMs. This study utilized a highly multiplexed immunoassay, the NULISAseq CNS panel, to compare plasma samples collected using BD‐P100 versus traditional EDTA tubes (EDTA) to evaluate the impact of tube types on biomarker profiles.

**Method:**

Venous blood was collected from participants in the Pittsburgh Alzheimer's Pathways Sleep Study (ALPS) at two visits, using both EDTA and BD‐P100 tubes. Plasma samples were obtained by centrifuging at 2000g for 10 minutes, then aliquoted and stored. The NULISAseq CNS Panel was performed on an Argo HT, following the manufacturer's instructions, using NULISA Protein Quantification (NPQ) values for biomarker quantification. Assay reproducibility was evaluated with a Sample Control (SC) run in triplicate across different runs. Spearman correlation assessed the concordance between different plasma samples.

**Result:**

This study included 116 plasma samples from 49 participants (aged 70.5 ± 4.1 years, 57% female, 90% non‐Hispanic White). NULISAseq robustly quantified 127 biomarkers, with median intra‐ and inter‐plate coefficients of variation (CVs) of 5.7% and 7.7%, respectively. High detectability was observed for both sample types, with medians of 94% (IQR: 2%) for EDTA and 96% (IQR: 3%) for BD‐P100. Most biomarkers showed a strong correlation between the two sample types, with a median Spearman coefficient (ρ) of 0.833 (IQR: 0.178). Classical AD biomarkers, including various tau forms and Aβ peptides, showed good concordance overall, ρ ranging from 0.540 to 0.942. Most biomarkers had similar NPQ levels in both sample types, with 86 biomarkers within a 10% deviation. HBA1 was significantly higher in BD‐P100, suggesting a higher level of hemolysis when BD‐P100 tubes were used for blood collection.

**Conclusion:**

Our results indicate that plasma collected with BD‐P100 and EDTA tubes have similar biomarker profiles. Further testing is needed to determine if BD‐P100 prolongs the stability of these biomarkers.

